# Etiologies of genital inflammation and ulceration in symptomatic Rwandan men and women responding to radio promotions of free screening and treatment services

**DOI:** 10.1371/journal.pone.0250044

**Published:** 2021-04-20

**Authors:** Kristin M. Wall, Julien Nyombayire, Rachel Parker, Rosine Ingabire, Jean Bizimana, Jeannine Mukamuyango, Amelia Mazzei, Matt A. Price, Marie Aimee Unyuzimana, Amanda Tichacek, Susan Allen, Etienne Karita

**Affiliations:** 1 Rwanda Zambia HIV Research Group, Department of Pathology & Laboratory Medicine, School of Medicine and Hubert Department of Global Health and Department of Epidemiology, Rollins School of Public Health, Laney Graduate School, Emory University, Atlanta, Georgia, United States of America; 2 Project San Francisco, Rwanda Zambia HIV Research Group, Kigali, Rwanda; 3 IAVI, NY, NY, University of California San Francisco, San Francisco, CA, United States of America; Universita degli Studi di Bologna Scuola di Medicina e Chirurgia, ITALY

## Abstract

**Introduction:**

The longstanding inadequacies of syndromic management for genital ulceration and inflammation are well-described. The Rwanda National Guidelines for sexually transmitted infection (STI) syndromic management are not yet informed by the local prevalence and correlates of STI etiologies, a component World Health Organization guidelines stress as critical to optimize locally relevant algorithms.

**Methods:**

Radio announcements and pharmacists recruited symptomatic patients to seek free STI services in Kigali. Clients who sought services were asked to refer sexual partners and symptomatic friends. Demographic, behavioral risk factor, medical history, and symptom data were collected. Genital exams were performed by trained research nurses and physicians. We conducted phlebotomy for rapid HIV and rapid plasma reagin (RPR) serologies and vaginal pool swab for microscopy of wet preparation to diagnose Trichomonas vaginalis (TV), bacterial vaginosis (BV), and vaginal Candida albicans (VCA). GeneXpert testing for Neisseria gonorrhoeae (NG) and Chlamydia trachomatis (CT) were conducted. Here we assess factors associated with diagnosis of NG and CT in men and women. We also explore factors associated with TV, BV and VCA in women. Finally, we describe genital ulcer and RPR results by HIV status, gender, and circumcision in men.

**Results:**

Among 974 men (with 1013 visits), 20% were positive for CT and 74% were positive for NG. Among 569 women (with 579 visits), 17% were positive for CT and 27% were positive for NG. In multivariate analyses, factors associated with CT in men included younger age, responding to radio advertisements, <17 days since suspected exposure, and not having dysuria. Factors associated with NG in men included not having higher education or full-time employment, <17 days since suspected exposure, not reporting a genital ulcer, and having urethral discharge on physical exam. Factors associated with CT in women included younger age and < = 10 days with symptoms. Factors associated with NG in women included younger age, lower education and lack of full-time employment, sometimes using condoms *vs*. never, using hormonal *vs*. non-hormonal contraception, not having genital ulcer or itching, having symptoms < = 10 days, HIV+ status, having BV, endocervical discharge noted on speculum exam, and negative vaginal wet mount for VCA. In multivariate analyses, only reporting >1 partner was associated with BV; being single and RPR+ was associated with TV; and having < = 1 partner in the last month, being pregnant, genital itching, discharge, and being HIV and RPR negative were associated with VCA. Genital ulcers and positive RPR were associated with being HIV+ and lack of circumcision among men. HIV+ women were more likely to be RPR+. In HIV+ men and women, ulcers were more likely to be herpetic rather than syphilitic compared with their HIV- counterparts.

**Conclusions:**

Syndromic management guidelines in Rwanda can be improved with consideration of the prevalence of confirmed infections from this study of symptomatic men and women representative of those who would seek care at government health centers. Inclusion of demographic and risk factor measures shown to be predictive of STI and non-STI dysbioses may also increase diagnostic accuracy.

## Introduction

Globally, over 1 million new sexually transmitted infections (STI) occur each day [1]. The prevalence of STI increased an estimated 59% in sub Saharan Africa between 1999 and 2005 and has continued to rise [2]. The World Health Organization (WHO) 2016–2021 Global Health Sector Strategy on Sexually Transmitted Infections aims to reduce STI 90% by 2030 using “[epidemiologic] information for focused action” [3].

The association between genital ulceration and inflammation (GUI) due to STI and non-STI etiologies and heterosexual HIV transmission and acquisition has been extensively studied in Africa [4–12]. Broadly, in observational studies GUI is associated with both transmitting and acquiring HIV in both men and women, and with transmission of more than one virion, an otherwise rare event, in cohabiting heterosexual discordant couples which comprise one of the largest HIV risk groups [6, 13–17].

Ulcerative STI that may facilitate HIV transmission include syphilis (*Treponema pallidum*, TP), Herpes simplex virus (HSV), and chancroid (*Haemophilus ducreyi*, HD) [18–20]. Inflammatory STI that increase HIV transmission include gonorrhea (*Neisseria gonorrhoeae*, NG), chlamydia (*Chlamydia trachomatis*, CT), and *Trichomonas vaginalis* (TV) [21–24]. Common non-STI dysbioses associated with genital inflammation include bacterial vaginosis (BV) and vaginal *Candida albicans (*VCA) [25–29].

Untreated TP, HD, HSV, NG, CT and TV can cause severe morbidity and, along with BV and VCA (which are troublesome but non-invasive), can contribute to HIV transmission. In our studies in African HIV discordant heterosexual couples, GUI contribute a substantial population attributable fraction of HIV transmission in both donor and recipient [15].

The longstanding inadequacies of syndromic management for GUI are well-described [30–37] but this approach remains the default in many resource-limited settings in Africa due to the high cost of molecular and culture-based diagnostics. The Rwanda National Guidelines for HIV and STI syndromic management were last updated in 2019 but these guidelines are not yet informed by the local prevalence and correlates of STI etiologies, a component WHO guidelines stress as critical to optimize locally relevant algorithms. We have previously published results of a survey of GUI among Female Sex Workers (FSW) in Kigali, but that study lacked molecular diagnostics for NG and CT [38].

Here we contribute to the epidemiologic data needed to inform improved diagnostic and treatment algorithms in Rwanda by exploring demographic, behavioral, medical history, symptom, genital exam, and laboratory factors associated with molecular diagnosis of NG and CT in men and women. We also explore factors associated with vaginal pathogens TV, BV and VCA in women. Finally, we describe genital ulcer and rapid plasma reagin (RPR) results stratified by gender, HIV status, and among men, by male circumcision status.

## Methods

### Ethics

This program was approved as non-research by the Rwandan National Ethics Committee. This program was determined to be non-research by the Emory Institutional Review Board criteria. Diagnostic and treatment were provided anonymously as free services.

### Setting

Kigali, the capital of Rwanda, has a population of over 1 million people and an adult HIV prevalence of 4.3% [39]. Between January 2016 and August 2019, The Center for Family Health Research (CFHR), a research site established in Kigali in 1986 and affiliated with Emory University in Atlanta, GA, USA, implemented a program for diagnosis and treatment of symptomatic GUI residents of Kigali. CFHR has worked closely with the Rwanda Ministry of Health (MoH) on research for improved HIV and reproductive health care in government-run health centers for many years [25, 40–43].

### Patient recruitment

Patients were residents of Kigali, Rwanda and were recruited in three ways: radio announcements, partner/friend referral, and pharmacist referral. Radio announcements were made in Kinyarwanda, Rwanda’s vernacular, encouraging men and women with symptoms suggestive of GUI (e.g., genital discharge, discomfort, ulcer) to seek free services at CFHR clinic and were broadcast throughout Kigali. Clients who sought services were then asked to refer sexual partners and symptomatic friends. Local pharmacists were alerted to the program and asked to refer individuals seeking treatments for suggestive symptoms. There were no inclusion/exclusion criteria applied to participant recruitment. Participants are representative of residents of Kigali with genital symptoms who self-selected to receive care.

### Data collection and diagnostic procedures

Demographics, behavioral risk factors, medical histories, and symptoms were collected using a standard instrument (S1 Fig). This information was obtained during interviews conducted by nurses who recorded data on paper and entered it into MS Access. Similarly, findings from genital exams performed by trained physicians and nurses were recorded on paper and entered into MS Access. Samples for laboratory testing were taken from all patients and included phlebotomy for rapid HIV and RPR serologies and vaginal pool swab for microscopy of wet preparation to diagnose TV, BV and VCA. GeneXpert testing for NG and CT (Cepheid, Sunnyvale USA) was conducted for all patients using endocervical swabs obtained from women and either urethral swabs (when discharge was reported or noted on physical exam) or urine samples from men. In collaboration with the MoH, CFHR developed a uniform alphanumeric identifier to allow anonymous data recording.

### Data analysis

Analyses were conducted with Statistical Analysis Software (SAS, Cary, NC). Frequencies of single and multiple infections were stratified by gender and HIV status. Demographic, behavioral, medical history, physical exam, microscopy and serology results were tabulated by gender and by NG and CT results. Bivariate and multivariate analyses of factors associated with NG or CT are presented in tables. Multivariable logistic regression models included variables associated with each outcome at p<0.05 in bivariate analysis and then backward selection was applied. Prevalence odds ratios (crude and adjusted, cPOR and aPOR, respectively) and 95% confidence intervals (CIs) and 2-sided p-values are presented. Variable multi-collinearity was assessed. Repeated visits by STI clients with new complaints were accounted for using the GENMOD procedure.

Bivariate and multivariate factors associated with vaginal pathogens TV, BV and VCA in women were analyzed in analogous fashion with results summarized in text. Demographic, behavioral, medical history, and HIV and RPR serology results were considered for model inclusion. Finally, genital ulcer and RPR results were described by gender, HIV status, and among men, by male circumcision status.

## Results

Unless specified in text, p-values are <0.05 for comparisons with details presented in Tables.

### Summary of GUI diagnosed in men and women (Table 1)

**Table 1:** Distribution of pathogens identified in symptomatic men and women in Kigali, Rwanda.

** **	**Total**		**HIV+ (N = 54)**		**HIV- (N = 958)**		**p-value**
	**N**	**Col %**	**N**	**Col %**	**N**	**Col %**	
Among all men (N = 1013 visits)*	** **	** **	** **	** **	** **	** **	
None identified	196	19%	14	26%	182	19%	0.210
CT	204	20%	7	13%	196	20%	0.181
NG	751	74%	40	74%	711	74%	0.981
CT and NG	138	14%	7	13%	131	14%	0.882
**Among men with RPR results (N = 975 visits)**			** **		** **		
None identified	184	19%	14	26%	170	18%	0.150
TP	52	5%	7	13%	45	5%	0.019
Any multiple infection	164	17%	11	21%	153	17%	0.433
** **	**Total**		**HIV+ (N = 75)**		**HIV- (N = 504)**		
**Among all women (N = 579 visits)**	**N**	**Col %**	**N**	**Col %**	**N**	**Col %**	
None identified	176	30%	13	17%	163	32%	0.008
CT	98	17%	8	11%	90	18%	0.121
NG	152	26%	34	45%	118	23%	<0.0001
CT and NG	45	8%	5	7%	40	8%	0.702
BV	113	21%	20	28%	93	19%	0.087
TV	72	13%	15	20%	57	12%	0.039
VCA	118	21%	6	8%	112	23%	0.004
**Among women with RPR results (N = 568 visits)**			** **		** **		
None identified	169	30%	13	18%	156	31%	0.020
TP	46	8%	16	22%	30	6%	<0.0001
Any multiple infection	146	26%	26	36%	120	24%	0.031

TP: *Treponema pallidum*, *NG*: *Neisseria gonorrhoeae*, CT: *Chlamydia trachomatis*, TV: *Trichomonas vaginalis*, *BV*: bacterial vaginosis, VCA: vaginal *Candida albicans*; RPR: rapid plasma regain

*One man missing HIV status

GeneXpert for NG and CT were provided to men during 1013 visits (974 unique men) between March 2017 and February 2019. Men tested HIV+ during 5% of these visits. Prevalence of NG was 74% and prevalence of CT was 20%, with no differences by HIV status. In the 975 visits with RPR results, TP prevalence was significantly higher among HIV+ (13%) compared with HIV- (5%) men. Nineteen percent of visits were negative for all pathogens, and 17% of visits had more than one infection identified.

GeneXpert for NG and CT were provided to women during 579 visits (569 unique women) between March 2017 and February 2019. Women tested HIV+ during 13% of these visits. Prevalence of NG was 26% and prevalence of CT was 17%, with higher prevalence of NG among HIV+ women. The prevalence of TV (overall 13%) was higher in HIV+ women, whereas the prevalence of VCA (overall 21%) was higher in HIV- women. In the 568 visits with RPR results, TP prevalence was significantly higher among HIV+ (22%) compared with HIV- (6%) women and having multiple pathogens identified was more prevalent among HIV+ (36%) compared with HIV- (24%) women’s visits. Conversely, having no pathogen identified was more prevalent in HIV- (31%) versus HIV+ (18%) women’s visits.

### Demographics and factors associated with CT and NG in men (Tables 2 and 3)

**Table 2:** Factors associated with CT or NG infection in men in Kigali, Rwanda (N = 1013).

	Total		CT-infected (N = 204)		CT-uninfected (N = 809)		p-value	NG-infected (N = 751)		NG-uninfected (N = 262)		p-value
	(N = 1013)											
Demographics	n /mean	Col% /SD	n /mean	Row% /SD	n /mean	Row% /SD		n /mean	Row% /SD	n /mean	Row% /SD	
**Age, continuous (years)**	30.8	7.1	29.4	5.6	31.1	7.4	0.001	30.5	7.0	31.6	7.3	0.029
**Referrer**	** **	** **										
Radio Advert	688	68%	151	22%	537	78%	0.037	488	71%	200	29%	0.001
Friends/Walk-in/Pharmacy/Contact Partner/Internet	325	32%	53	16%	272	84%		263	81%	62	19%	
**Living and Marital Status**	** **	** **										
Married and Cohabiting	232	23%	33	14%	199	86%	0.011	156	67%	76	33%	0.006
Single or Divorced/Separated/Widow	781	77%	171	22%	610	78%		595	76%	186	24%	
**Education Level**	** **	** **										
None	25	2%	1	4%	24	96%	0.095	16	64%	9	36%	0.001
Primary	339	34%	66	19%	273	81%		267	79%	72	21%	
Secondary	454	45%	89	20%	365	80%		343	76%	111	24%	
Higher	193	19%	47	24%	146	76%		123	64%	70	36%	
**Employment Status**	** **	** **										
Full-time employment	552	55%	122	22%	430	78%	0.095	392	71%	160	29%	0.015
Part-time/Student/Jobless	459	45%	82	18%	377	82%		357	78%	102	22%	
**Sexual behaviors**	** **	** **	** **	** **	** **	** **	** **	** **	** **	** **	** **	** **
**Number of partners in last 30 days**	** **	** **										
None or one partner	704	78%	138	20%	566	80%	0.422	518	74%	186	26%	0.051
More than one partner	203	22%	45	22%	158	78%		163	80%	40	20%	
**Condom use during vaginal sex in the last three months**												
No partners or always used condoms	27	3%	4	15%	23	85%	0.555	14	52%	13	48%	0.015
Sometimes	363	40%	69	19%	294	81%		279	77%	84	23%	
Never	517	57%	110	21%	407	79%		387	75%	130	25%	
**Number of days since sexual contact you suspect STI was acquired from**												
< = 8	331	35%	83	25%	248	75%	0.010	292	88%	39	12%	<0.0001
9–16	288	31%	58	20%	230	80%		235	82%	53	18%	
> = 17	323	34%	50	15%	273	85%		177	55%	146	45%	
**Self-reported symptoms**	** **	** **	** **	** **	** **	** **	** **	** **	** **	** **	** **	** **
**Urethral discharge**												
Yes	895	89%	188	21%	707	79%	0.081	717	80%	178	20%	<0.0001
No	114	11%	16	14%	98	86%		32	28%	82	72%	
**Dysuria**												
Yes	810	80%	153	19%	657	81%	0.034	599	74%	211	26%	0.680
No	199	20%	51	26%	148	74%		150	75%	49	25%	
**Genital itching**												
Yes	67	7%	14	21%	53	79%	0.864	39	58%	28	42%	0.001
No	854	93%	171	20%	683	80%		649	76%	205	24%	
**Genital ulcer**												
Yes	41	4%	6	15%	35	85%	0.336	13	32%	28	68%	<0.0001
No	878	96%	183	21%	695	79%		681	78%	197	22%	
**Number of days with symptoms**												
1–5	385	41%	100	26%	285	74%	0.004	332	86%	53	14%	<0.0001
6–10	254	27%	40	16%	214	84%		191	75%	63	25%	
11–21	192	21%	33	17%	159	83%		121	63%	71	37%	
>21	105	11%	17	16%	88	84%		56	53%	49	47%	
**Laboratory and physical exam**	** **	** **	** **	** **	** **	** **	** **	** **	** **	** **	** **	** **
**HIV Status**												
Positive	54	5%	7	13%	47	87%	0.181	40	74%	14	26%	0.981
Negative	958	95%	196	20%	762	80%		711	74%	247	26%	
**RPR Result**												
Positive	52	5%	13	25%	39	75%	0.354	43	83%	9	17%	0.136
Negative	923	95%	182	20%	741	80%		677	73%	246	27%	
**Urethral discharge**												
Yes	858	91%	178	21%	680	79%	0.199	692	81%	166	19%	<0.0001
No	87	9%	13	15%	74	85%		15	17%	72	83%	
**Genital ulcer**												
Yes	46	5%	8	17%	38	83%	0.623	17	37%	29	63%	<0.0001
No	898	95%	183	20%	715	80%		686	76%	212	24%	
**Circumcision status**												
Circumcised	524	67%	122	23%	402	77%	0.058	416	79%	108	21%	0.192
Uncircumcised	259	33%	45	17%	214	83%		195	75%	64	25%	

Not significant not shown include: Self-reported symptoms dyspareunia, unpleasant odor, abdominal pain, anal discharge, anal ulcer, anal warts, and sore throat; genital exam results white accumulation, condyloma/warts, inguinal adenopathy >1cm unilateral and bilateral, inflammation, and testicular mass/tenderness

RPR: Rapid plasma reagin; STI: Sexually transmitted disease; NG: Neisseria gonorrhoeae, CT: Chlamydia trachomatis

**Table 3:** Univariate and multivariate analysis of factors associated with CT or NG infection in men in Kigali, Rwanda (N = 1013).

	CT infection								NG infection							
Demographics	cPOR	95% CI		p-value	aPOR	95% CI		p-value	cPOR	95% CI		p-value	aPOR	95% CI		p-value
**Age (per year increase)**	0.96	0.94	0.99	0.001	0.96	0.94	0.98	0.001	0.98	0.96	1.00	0.029				
**Referrer**	** **	** **							** **	** **						
Radio Advert	1.44	1.02	2.04	0.038	1.44	1.01	2.07	0.046	ref	---	---	---				
Friends/Walk-in/Pharmacy/Contact Partner/Internet	ref				ref				1.76	1.28	2.43	0.001				
**Living and Marital Status**	** **								** **							
Married and Cohabiting	ref								ref	---	---	---				
Single or Divorced/Separated/Widow	1.69	1.13	2.54	0.011					1.56	1.14	2.15	0.006				
**Education Level**	** **								** **							
None/Primary/Secondary	ref								1.84	1.32	2.58	0.000	2.39	1.57	3.63	<0.0001
Higher	1.37	0.95	1.99	0.092					ref	---	---	---	ref	---	---	---
**Employment Status**	** **								** **							
Full-time employment	1.30	0.96	1.78	0.094					ref	---	---	---	ref	---	---	---
Part-time/Student/Jobless	ref								1.45	1.09	1.92	0.011	1.51	1.05	2.17	0.028
**Sexual behaviors**	** **	** **	** **	** **	** **	** **	** **	** **								
**Number of partners in last 30 days**	** **	** **							** **	** **						
None or one partner	ref								ref	---	---	---				
More than one partner	1.17	0.80	1.71	0.424					1.5	1.02	2.2	0.040				
**Condom use during vaginal sex in the last 3 months**																
No partners or always used condoms	0.64	0.22	1.90	0.426					0.37	0.17	0.8	0.012				
Sometimes	0.87	0.62	1.21	0.411					1.13	0.83	1.54	0.450				
Never	ref								ref	---	---	---				
**Number of days since sexual contact you suspect STI was acquired from**																
0–16	1.61	1.13	2.30	0.009	1.64	1.15	2.35	0.007	4.68	3.43	3.37	<0.0001	3.29	2.30	4.7	<0.0001
> = 17	ref				ref				ref	---	---	---	ref	---	---	---
**Self-reported symptoms**	** **	** **	** **	** **	** **	** **	** **	** **								
**Urethral discharge**																
Yes	1.63	0.94	2.83	0.084					10.00	6.41	15.61	<0.0001				
No	ref								ref	---	---	---				
**Dysuria**																
Yes	ref				ref				ref	---	---	---				
No	1.48	1.03	2.13	0.034	1.51	1.03	2.22	0.035	1.05	0.74	1.49	0.792				
**Genital itching**																
Yes	1.05	0.57	1.93	0.872					ref	---	---	---				
No	ref								2.24	1.35	3.72	0.002				
**Genital ulcer**																
Yes	ref								ref	---	---	---	ref	---	---	---
No	1.53	0.63	3.70	0.345					7.50	3.79	14.85	<0.0001	4.50	2.22	9.13	<0.0001
**Number of days with symptoms**																
1–10	1.39	0.97	1.98	0.075					3.06	2.26	4.15	<0.0001				
> = 11	ref								ref	---	---	---				
**Laboratory and physical exam**	** **	** **	** **	** **	** **	** **	** **	** **								
**HIV Status**																
Positive	0.58	0.26	1.31	0.189					0.99	0.53	1.86	0.980				
Negative	ref								ref	---	---	---				
**RPR Result**																
Positive	1.30	0.68	2.52	0.429					1.65	0.82	3.3	0.158				
Negative	ref								ref	---	---	---				
**Urethral discharge**																
Yes	1.49	0.81	2.75	0.204					19.94	11.12	35.76	<0.0001	16.38	7.28	36.89	<0.0001
No	ref								ref	---	---	---	ref	---	---	---
**Genital ulcer**																
Yes	0.82	0.38	1.80	0.626					ref	---	---	---				
No	ref								5.52	2.96	10.28	<0.0001				

aPOR: Adjusted prevalence odds ratio; cPOR: Crude prevalence odds ratio; RPR: Rapid plasma reagin; CI: Confidence interval; STI: Sexually transmitted disease; NG: Neisseria gonorrhoeae, CT: Chlamydia trachomatis

Not significant not shown include: Self-reported symptoms dyspareunia, unpleasant odor, abdominal pain, anal discharge, anal ulcer, anal warts, and sore throat; genital exam results white accumulation, condyloma/warts, inguinal adenopathy >1cm

Men averaged 30.8 years of age, 77% were single, 64% had at least a secondary education, 55% were employed full time, 22% reported more than one partner in the last 30 days and 57% reported never using condoms in the past three months. The most common symptoms reported were urethral discharge (89%) and dysuria (80%). Physical findings included urethral discharge in 91% and genital ulcer in 5% of men (Table 2).

Multivariate analyses (Table 3) showed younger age, responding to radio advertisements, <17 days since suspected exposure, and not having dysuria as independent factors associated with CT.

Multivariate analyses (Table 3) showed not having higher education or full-time employment, <17 days since suspected exposure, not reporting a genital ulcer, and urethral discharge on physical exam as independent factors associated with NG.

HIV, RPR serologic results, and circumcision status were not associated with either CT or NG.

### Demographics and factors associated with CT and NG in women (Tables 4 and 5)

**Table 4:** Factors associated with CT or NG infection in women in Kigali, Rwanda (N = 579).

	Total (N = 579)		CT-infected (N = 98)		CT-uninfected (N = 481)		p-value	NG-infected (N = 152)		NG-uninfected (N = 427)		p-value
Demographics	n /mean	Col%/SD	n /mean	Row%/SD	n /mean	Row% /SD		n /mean	Row%/SD	n /mean	Row%/SD	
**Age, continuous (years)**	28.7	7.2	25.6	6.1	29.3	7.2	<0.0001	26.8	6.3	29.4	7.4	<0.0001
**Referrer**	** **	** **										
Radio Advert	284	49%	37	13%	247	87%	0.014	67	24%	217	76%	0.153
Friends/Walk-in/Pharmacy/Contact Partner/Internet	295	51%	61	21%	234	79%		85	29%	210	71%	
**Living and Marital Status**	** **	** **										
Married and Cohabiting	268	46%	34	13%	234	87%	0.012	60	22%	208	78%	0.050
Single or Divorced/Separated/Widow	311	54%	64	21%	247	79%		92	30%	219	70%	
**Education Level**	** **	** **										
None	25	4%	2	8%	23	92%	0.513	9	36%	16	64%	0.001
Primary	242	42%	38	16%	204	84%		81	33%	161	67%	
Secondary	246	42%	46	19%	200	81%		54	22%	192	78%	
Higher	66	11%	12	18%	54	82%		8	12%	58	88%	
**Employment Status**	** **	** **										
Full-time employment	199	34%	30	15%	169	85%	0.383	39	20%	160	80%	0.008
Part-time/Student/Jobless	379	66%	68	18%	311	82%		113	30%	266	70%	
**Sexual behaviors**	** **	** **	** **	** **	** **	** **	** **	** **	** **	** **	** **	** **
**Number of partners in last 30 days**	** **	** **										
None or one partner	444	83%	70	16%	374	84%	0.112	95	21%	349	79%	<0.0001
More than one partner	88	17%	20	23%	68	77%		43	49%	45	51%	
**Condom use during vaginal sex in the last 3 months**												
No partners or always used condoms	35	7%	6	17%	29	83%	0.259	4	11%	31	89%	<0.0001
Sometimes	163	31%	34	21%	129	79%		66	40%	97	60%	
Never	334	63%	50	15%	284	85%		68	20%	266	80%	
**Number of days since sexual contact you suspect STI was acquired from**												
< = 8	46	9%	8	17%	38	83%	0.066	15	33%	31	67%	0.003
9–16	78	15%	20	26%	58	74%		31	40%	47	60%	
> = 17	409	77%	61	15%	348	85%		92	22%	317	78%	
**Number of children under 18, continuous**	1.3	1.3	1.0	1.1	1.3	1.3	0.026	1.2	1.1	1.3	1.3	0.600
**Number of additional children desired, continuous**	1.4	1.1	1.6	1.1	1.4	1.2	0.040	1.3	1.0	1.4	1.2	0.279
**Pregnant**												
Yes	48	8%	8	17%	40	83%	0.947	11	23%	37	77%	0.569
No	528	92%	90	17%	438	83%		141	27%	387	73%	
**Want more children in next two years**												
Yes	125	23%	20	16%	105	84%	0.666	33	27%	88	73%	0.821
No	419	77%	74	18%	345	82%		106	26%	298	74%	
**Family planning method among women not pregnant and do not want more children in next two years**												
Non-Hormonal Method (IUD/Condom/Tubal Ligation/Natural Method) or No Method	268	66%	47	18%	221	82%	0.498	56	21%	212	79%	0.001
Hormonal Implant	50	12%	9	18%	41	82%		24	48%	26	52%	
Injectable	48	12%	5	10%	43	90%		16	33%	32	67%	
Pills	40	10%	9	23%	31	78%		12	30%	28	70%	
**Family planning method and pregnancy composite**												
Pregnant	48	8%	8	17%	40	83%	0.979	11	23%	37	77%	0.003
Hormonal method (implant, injectable, pills)	139	24%	23	17%	116	83%		52	37%	87	63%	
Non-Hormonal (IUD/Condom/ Tubal Ligation/Natural Method) or No Method	388	67%	67	17%	321	83%		89	23%	299	77%	
**Self-reported symptoms**	** **	** **	** **	** **	** **	** **	** **	** **	** **	** **	** **	** **
**Vaginal discharge**												
Yes	475	82%	78	16%	397	84%	0.412	123	26%	352	74%	0.704
No	101	18%	20	20%	81	80%		28	28%	73	72%	
**Genital itching**												
Yes	320	56%	52	16%	268	84%	0.732	57	18%	263	82%	<0.0001
No	254	44%	44	17%	210	83%		92	36%	162	64%	
**Dysuria**												
Yes	266	46%	44	17%	222	83%	0.793	75	28%	191	72%	0.306
No	311	54%	54	17%	257	83%		76	24%	235	76%	
**Genital ulcer**												
Yes	64	11%	9	14%	55	86%	0.489	9	14%	55	86%	0.020
No	508	89%	89	18%	419	82%		140	28%	368	72%	
**Number of days with symptoms**												
1–5	72	13%	16	22%	56	78%	0.170	24	33%	48	67%	0.003
6–10	77	14%	17	22%	60	78%		27	35%	50	65%	
11–21	131	24%	18	14%	113	86%		40	31%	91	69%	
>21	257	48%	37	14%	220	86%		48	19%	209	81%	
**Laboratory and physical exam**	** **	** **	** **	** **	** **	** **	** **	** **	** **	** **	** **	** **
**HIV Status**												
Positive	75	13%	8	11%	67	89%	0.121	34	45%	41	55%	<0.0001
Negative	504	87%	90	18%	414	82%		118	23%	386	77%	
**RPR Result**												
Positive	46	8%	10	22%	36	78%	0.401	21	46%	25	54%	0.002
Negative	522	92%	88	17%	434	83%		128	25%	394	75%	
**Trichomonas**												
Positive	72	13%	18	25%	54	75%	0.038	18	25%	54	75%	0.818
Negative	491	87%	75	15%	416	85%		129	26%	362	74%	
**Candida**												
Positive	118	21%	12	10%	106	90%	0.035	13	11%	105	89%	<0.0001
Negative	437	79%	80	18%	357	82%		132	30%	305	70%	
**Bacterial vaginosis**												
Positive	113	21%	25	22%	88	78%	0.062	47	42%	66	58%	<0.0001
Negative	438	79%	65	15%	373	85%		96	22%	342	78%	
**Vaginal Inflammation or Discharge**												
Yes	469	87%	75	16%	394	84%	0.232	116	25%	353	75%	0.076
No	69	13%	15	22%	54	78%		24	35%	45	65%	
**Endocervical Inflammation or Discharge**												
Yes	262	49%	55	21%	207	79%	0.010	88	34%	174	66%	<0.001
No	275	51%	35	13%	240	87%		52	19%	223	81%	
**Genital Ulcer**												
Yes	48	9%	9	19%	39	81%	0.652	12	25%	36	75%	0.857
No	481	91%	78	16%	403	84%		126	26%	355	74%	

Not significant not shown include: Self-reported symptoms anal discharge, anal ulcer, anal warts, and sore throat; genital exam results non-menstrual bleeding (cervix and vagina), condyloma/warts (cervix and vagina), inguinal adenopathy >1cm unilateral and bilateral, adnexal tenderness and adnexal mass.

IUD: intrauterine device; RPR: Rapid plasma reagin; CI: Confidence interval; STI: Sexually transmitted disease; NG: Neisseria gonorrhoeae, CT: Chlamydia trachomatis

**Table 5:** Univariate and multivariate analysis of factors associated with CT or NG infection in women in Kigali, Rwanda (N = 579).

	CT infection								NG infection							
	cPOR	95% CI		p-value	aPOR	95% CI		p-value	cPOR	95% CI		p-value	aPOR	95% CI		p-value
**Demographics**	** **	** **	** **	** **	** **	** **	** **	** **	** **	** **	** **	** **	** **	** **	** **	** **
**Age (per year increase)**	0.91	0.88	0.95	<0.0001	0.90	0.86	0.94	<0.0001	0.95	0.92	0.97	< .001	0.93	0.89	0.97	<0.001
**Referrer**	** **	** **							** **	** **						
Radio Advert	ref								ref							
Friends/Walk-in/Pharmacy/Contact Partner/Internet	1.74	1.11	2.72	0.015					1.31	0.91	1.90	0.150				
**Living and Marital Status**	** **								** **							
Married and Cohabiting	ref								ref							
Other	1.78	1.13	2.80	0.012					1.46	1.00	2.13	0.048				
**Education Level**	** **								** **							
None/Primary	ref								2.09	1.44	3.04	0.000	2.13	1.30	3.48	0.003
Secondary/Higher	1.30	0.83	2.01	0.248					ref				ref			
**Employment Status**	** **								** **							
Full-time employment	ref								ref				ref			
Part-time/Student/Jobless	1.23	0.77	1.96	0.383					1.76	1.16	2.66	0.008	1.95	1.12	3.39	0.019
**Sexual behaviors**	** **	** **	** **	** **	** **	** **	** **	** **	** **	** **	** **	** **	** **	** **	** **	** **
**Number of partners in last 30 days**	** **	** **							** **	** **						
None or one partner	ref								ref							
More than one partner	1.56	0.89	2.75	0.119					3.53	2.19	5.69	<0.0001				
**Condom use during vaginal sex in the last 3 months**																
No partners or always used condoms	1.17	0.46	2.97	0.741					0.48	0.15	1.5	0.207	0.74	0.22	2.41	0.611
Sometimes	1.49	0.92	2.42	0.107					2.75	1.81	4.18	<0.0001	1.79	1.07	2.98	0.025
Never	ref								ref				ref			
**Number of days since sexual contact you suspect STI was acquired from**																
0–8	1.20	0.53	2.69	0.666					1.66	0.87	3.16	0.126				
9–16	1.96	1.1	3.49	0.022					2.29	1.36	3.87	0.002				
> = 17	ref								ref							
**Number of children under 18 (per child increase)**	0.82	0.69	0.99	0.037					0.96	0.84	1.10	0.594				
**Number of additional children desired (per child increase)**	1.22	1.02	1.45	0.027					0.92	0.79	1.07	0.274				
**Family planning method and pregnancy composite**																
Pregnant	0.96	0.43	2.14	0.915					1.00	0.49	2.03	0.999	1.30	0.57	2.99	0.532
Hormonal method (implant, injectable, pills)	0.95	0.56	1.59	0.837					2.01	1.32	3.05	0.001	1.73	1.02	2.94	0.040
Non-Hormonal (IUD/Condom/Tubal Ligation/Natural Method) or No Method	ref								ref				ref			
**Self-reported symptoms**	** **	** **	** **	** **	** **	** **	** **	** **	** **	** **	** **	** **	** **	** **	** **	** **
**Vaginal discharge**																
Yes	ref								ref							
No	1.26	0.73	2.18	0.408					1.10	0.68	1.78	0.692				
**Genital itching**																
Yes	ref								ref				ref			
No	1.08	0.69	1.68	0.738					2.62	1.79	3.84	<0.0001	2.54	1.55	4.17	0.0002
**Dysuria**																
Yes	ref								1.21	0.84	1.75	0.303				
No	1.06	0.68	1.64	0.796					ref							
**Genital ulcer**																
Yes	ref								ref				ref			
No	1.30	0.62	2.73	0.489					2.33	1.12	4.84	0.024	2.52	1.09	5.80	0.030
**Number of days with symptoms**																
1–10	1.72	1.06	2.78	0.027	1.76	1.07	2.88	0.026	1.76	1.16	2.68	0.008	1.78	1.05	3.00	0.032
11 or more	ref				ref				ref				ref			
**Laboratory and physical exam**	** **	** **	** **	** **	** **	** **	** **	** **	** **	** **	** **	** **	** **	** **	** **	** **
**HIV Status**																
Positive	ref								2.73	1.66	4.47	<0.0001	2.05	1.10	3.83	0.024
Negative	1.83	0.85	3.96	0.124					ref				ref			
**RPR Result**																
Positive	1.37	0.66	2.88	0.401					2.58	1.41	4.7	0.002				
Negative	ref								ref							
**Trichomonas**																
Positive	1.85	1.03	3.32	0.041					ref							
Negative	ref								1.06	0.60	1.88	0.838				
**Candida**																
Positive	ref								ref				ref			
Negative	1.98	1.04	3.77	0.038					3.56	1.89	6.69	<0.0001	2.20	1.11	4.36	0.024
**Bacterial vaginosis**																
Positive	1.63	0.98	2.72	0.063					2.63	1.67	4.15	<0.0001	1.89	1.07	3.34	0.028
Negative	ref								ref				ref			
**Vaginal Inflammation OR Discharge**																
Yes	ref								ref							
No	1.47	0.79	2.75	0.2201					1.67	0.97	2.86	0.063				
**Endocervical Inflammation or Discharge**																
Yes	1.83	1.15	2.91	0.010					2.17	1.46	3.23	0.000	1.80	1.11	2.93	0.018
No	ref								ref				ref			
**Genital Ulcer**																
Yes	1.19	0.56	2.56	0.649					ref							
No	ref								1.06	0.53	2.10	0.875				

IUD: intrauterine device; aPOR: Adjusted prevalence odds ratio; cPOR: Crude prevalence odds ratio; RPR: Rapid plasma reagin; CI: Confidence interval; STI: Sexually transmitted disease; NG: Neisseria gonorrhoeae, CT: Chlamydia trachomatis

The mean age women was 28.7, they had 1.3 children and desired 1.4 more on average, 54% were single, 53% had a secondary education or more, 34% had full-time employment, 83% reported < = 1 partner in the last 30 days and 63% reported never using condoms in the past three months. Vaginal discharge was the most common presenting symptom (82%) and endocervical inflammation or discharge was noted on 49% of speculum exams. (Table 4)

Multivariate analyses (Table 5) showed younger age and having symptoms < = 10 days as independent factors associated with CT.

Multivariate analyses (Table 5) showed younger age, lower education and lack of full-time employment, sometimes using condoms *vs*. never, using hormonal contraception *vs*. other or no contraception, not having a genital ulcer or itching, having symptoms for < = 10 days, HIV+ status, endocervical discharge noted on speculum exam, BV, and negative VCA as independent factors associated with NG.

### Factors associated with of BV, TV and VCA in women (not tabled)

Only reporting >1 partner remained independently associated with BV in multivariate analyses (POR 2.21, p = 0.003). Factors associated with TV in multivariate analyses were being single and RPR+ (aPOR 2.05, p = 0.009 and aPOR 2.37, p = 0.023, respectively). Factors associated with VCA were having < = 1 partner in the last month (aPOR 4.26, p = 0.005), being pregnant (aPOR 3.05, p = 0.002), always using condoms or not having sex in the last three months *vs*. never using condoms (aPOR 2.42, p = 0.023), genital itching (aPOR 1.69, p = 0.034), genital discharge (aPOR 2.56, p = 0.011), and being HIV and RPR negative (aPOR 2.93, p = 0.025 and aPOR 4.94, p = 0.031, respectively).

### Genital ulcers in men and women (not tabled)

Reported and/or observed genital ulcers were more common among HIV+ (20%) compared with HIV- (5%) men (p<0.001). Genital ulcers were noted during physical examination in 19% of RPR+ and 4% of RPR- men and conversely 20% of men with ulcers were RPR+ compared to 4% of men without ulcers (p<0.001). Among HIV+ men, none of the seven who were RPR+ had reported and/or observed ulcers while 23% of 43 HIV+ RPR- men had ulcers (p = 0.319). In contrast, among HIV- RPR+ men 21% had reported or observed ulcers compared to only 4% of HIV-RPR- men (p<0.001). This suggests that ulcers among HIV+ men were more likely herpetic while among HIV- men at least one fifth were syphilitic.

Although HIV- men were more likely to be circumcised than HIV+ men (67% *vs*. 58%) in our program, this difference was not significant (p = 0.196). Among circumcised men, those who were HIV+ were more likely to have ulcers (13% *vs*. 4%, p = 0.074) and to be RPR+ (20% *vs*. 4%, p = 0.003). Among uncircumcised men, those who were HIV+ were also more likely to have ulcers (27% *vs*. 7%, p = 0.001) while the difference in RPR+ results was not significant (12% *vs*. 6%, p = 0.324).

Among women, the prevalence of reported or observed ulcers was not significantly different by HIV serostatus (20% in HIV+ vs.14% p = 0.196). Genital ulcers were noted during physical examination for 28% of RPR+ women compared with 14% of RPR- women (p<0.001). As with men, the association between RPR results and reported and/or observed ulcers differed in HIV+ and HIV- women: 25% of HIV+RPR+ *vs*. 20% of HIV+RPR- had ulcers, p = 0.729, compared with 37% of HIV-RPR+ vs. 13% of HIV-RPR- women having ulcers (p = 0.001).

## Discussion

We found a high prevalence of NG and CT among symptomatic men and women in Kigali. Among men, urethral discharge was strongly associated with a diagnosis of NG while dysuria was not associated with either infection. Specific symptoms were less helpful in identifying NG and CT among women. Physical exam findings, demographic variables and reported risk behaviors were independently predictive of NG and/or CT in both men and women, as were vaginal wet mount findings and HIV serologies among women. Among women, TV and BV were associated with sexual risk behaviors but not with symptoms while VCA was associated with vaginal itching and discharge and with low-risk profiles. There were complex inter-relationships between HIV and RPR serologies and genital ulcers, and these were further influenced by circumcision status among men. These findings exemplify the locally relevant data that can inform approaches to diagnosis and treatment in Rwanda as called for by WHO. Our models had good discrimination and use of these data may offer improvement over the current algorithm recommended by the Rwandan National Guidelines.

As in other studies, syndromic management may perform better among men compared to women due to the ease of detecting abnormalities on external genitalia and the high likelihood of NG among men reporting urethral discharge [44]. Surprisingly, dysuria was as common as discharge in men but contrary to conventional wisdom we did not find an association between dysuria and NG or CT [45].

The most common presenting symptom among women was vaginal discharge which was only associated with VCA and not with NG, CT, BV or TV. Genital itching was reported by over half of patients and was also predictive of VCA. Itching was also useful in pointing away from NG, as was reported ulcer. Gynecologic exam, specifically endocervical discharge, was helpful in the diagnosis of NG. Interestingly, wet mount results were predictive NG (BV+, VCA-), suggesting that these inexpensive and simple tests should be included in any workup of symptomatic women. Despite extensive laboratory testing, we failed to find an etiology for a substantial proportion of women seeking care. This may reflect poor sensitivity of microscopy as well as non-infectious causes of symptoms. As has been noted elsewhere, factors associated with NG were more useful in predicting infections than those for CT [46, 47].

For both men and women, younger age was predictive of both NG and CT and lower education level and jobless or part-time employment status were predictive of NG. Interestingly, number of partners was not independently associated with CT or NG. Most men and women reported never using condoms and very few reported always using condoms. Women who sometimes used condoms were at higher risk of NG than those who never used them. This may be due to increased condom use in women with higher risk partners.

Genital ulcers were not a common presenting symptom and were not associated with RPR results among HIV+ patients. RPR provided a diagnosis for 20% of ulcers among HIV- men and 15% among HIV- women. As others in Africa have reported, HSV is the most likely diagnosis for RPR- ulcers which was more common among HIV+ patients [48]. Non-circumcision among men is associated with HIV acquisition and with increased prevalence and incidence of ulcerative STI [49–52]. We have previously shown a relationship between ulcers, smegma and HIV acquisition in uncircumcised men [15]. Among HIV- men, those who were uncircumcised were not more likely to report ulcers or to be RPR+ than their circumcised counterparts. In contrast, among HIV+ men, those who were uncircumcised were more likely to have an ulcer and less likely to be RPR+ than circumcised men. Circumcision is widely promoted in Rwanda and available at no cost in most government health centers as part of HIV prevention services. Though the focus is on protecting HIV- men, our results here suggest that circumcision can benefit HIV+ men by reducing ulcer incidence [53].

It is likely that we missed other less common ulcer etiologies including HD, lymphogranuloma venereum (LGV), and granuloma inguinale (*Klebsiella granulomatis*) [54]. Our clinicians did suspect chancroid in a few cases, but the service program did not record detailed descriptions or photographs of ulcers and we lacked laboratory diagnostics. The most recent publication presenting confirmed chancroid diagnoses in Rwanda was based on data collected in 1992, which found 27% of ulcers in men and 20% in women had culture-confirmed HD [55–59]. For many years the prevalence of HD had been decreasing in much of Africa [48, 54], but recent publications indicate HD may be staging a comeback [21]. More investigations are needed in Rwanda.

Physical exam findings made important contributions in our program. Examination of male genitalia does not require specialized equipment, but speculum exam requires a skilled clinician, a gynecologic exam table and light which are in limited supply in low resource settings. While genital exams would not be feasible for all symptomatic patients, targeted genital exams in specific circumstances would be feasible and potentially very useful. Distinguishing between vaginal and endocervical discharges would greatly improve diagnostic accuracy and bi-manual exam would identify pelvic inflammatory disease. Similarly, in our setting where less than one in five ulcer patients are RPR+, assessing ulcer characteristics may be worthwhile. Visual exam has traditionally been viewed as unreliable as many ulcers do not have a paradigmatic presentation (e.g. painless ‘clean’ TP ulcer, painful ‘dirty’ HD with inguinal adenopathy, multiple chronic or recurrent shallow vesicular HSV lesions). However, a recent study in Jamaica compared clinical diagnosis with M-PCR and found visual diagnoses of TP, HSV, and HD were 67.7%, 53.8%, and 75% sensitive and 91.2%, 83.6%, and 75.4% specific, respectively [60].

The advent of point-of-care diagnostics for NG and CT has transformed STI diagnosis, but given relatively expensive equipment and reagents, this remains out of reach in many low resource settings. We have used pooling to reduce the per-patient cost in Zambia and this could be explored in other settings [61]. GeneXpert kits are also available for TV and they are more sensitive than microscopy. The US CDC has in-house multiplex PCR (M-PCR) for ulcer etiologies including syphilis, HSV and chancroid. A focused study would provide prevalence information that could inform the next update of national guidelines.

Our program has several limitations. Social desirability bias may have led to under-reporting of risky sexual behaviors. We focused on symptomatic men and women and thus missed the many people who are asymptomatically infected [62, 63]. We did not screen for active viral hepatitis as recent unpublished surveys have shown a low prevalence of both hepatitis B and C (4% and 3%, respectively reported nationally, 4% and 5% among female sex workers tested in our laboratory). We did not have funding or resources to perform any direct method of detection for TP using ulcer material, and thus may have misclassified some recently infected people who were negative by RPR test. While we did treat TV in male partners referred by TV+ women, we did not systematically test for TV in men. Microscopy for TV detection in men is extremely insensitive, and we did not have resources to conduct GeneXpert testing for TV. TV could therefore be the reason for a portion of the symptomatic men with unknown etiology. We did not include HSV serologies because adult seroprevalence is high [64]. Assessment of cervical intraepithelial neoplasia requires more resources than would be achievable on a large scale in Rwandan health centers so we did not address this important problem. Fortunately, 93% of Rwandan girls now receive the human papillomavirus vaccine and future generations will be protected [65]. Lastly, we and others have published an association between female genital schistosomiasis and HIV [66, 67], but this is most commonly seen with *S*.*Haematobium* while only *S*.*Mansoni* is endemic in Rwanda, thus we did not screen for genital schistosomiasis [68].

## Conclusions

Syndromic management guidelines in Rwanda can be improved with consideration of the prevalence of confirmed infections from this program offering services to symptomatic men and women representative of those who would seek care at government health centers. Our findings indicate that syndromic management performs better among men but is poor among women. Inclusion of demographic and risk factor measures shown to be predictive of STI and non-STI dysbioses may also increase diagnostic accuracy. In symptomatic women, wet mount results for BV and VCA may help diagnose NG and are inexpensive and could be offered for management of women. Targeted genital exams for women in specific circumstances (e.g., in women without genital itching) may also be useful to diagnose NG. More data is needed on how often local prevalence and epidemiology should be reassessed to maintain improved syndromic management.

## Supporting information

S1 Fig STI baseline clinical form. (DOCX)
