# Supplementary material for: Etiologies of genital inflammation and ulceration in symptomatic Rwandan men and women responding to radio promotions of free screening and treatment services
Source: PLoS One. 2021 Apr 20;16(4):e0250044. doi: 10.1371/journal.pone.0250044 (PMC8057583; doi:10.1371/journal.pone.0250044)
Supplement: S1 Fig — (DOCX) [file pone.0250044.s001.docx]

**S1 Fig.** Questionnaire administered to STI service participants

**Part I: Socio-Demographic**

| **ID(Code):**  **Client oriented by:**   - Radio advert - Pharmacy - Invitation/ Contact partner | - Heard from friends/walkin - Internet - OPD - Other _____________________ | | | **Participant Type:**   - STI Female Patient - STI Male Patient - VMMC   **Date of Visit:**  **--------/--------/--------**  **(Day/Month/Year)** |
| --- | --- | --- | --- | --- |
| Imibereho (Living Situation)   - Aribana (Live alone) - Abana n’ababyeyi/Umuryango (Live with parents/family members) - Abana n’umugabo (Cohabiting) - Abana n’inshuti ze (Live with roommates) - Ibindi (Other) | | Marital Status   - Ingaragu (Single) - Arubatse (Married) - Yatandukanye n’umugabo (Divorced/Separated) - Umupfakazi (Widow) | | |
| Amashuri yize (Education)   - Ntiyigeze yiga - None - Abanza, Imyaka _____ (Primary) - Ayisumbuye, imyaka ________ (Secondary) - Kaminuza (Higher) | | | Ufite akazi uhemberwa? (Are you employed at a paid job?)   - Yego, ndakora (Yes, full-time) - Yego, ibiraka (Yes, part-time) - Oya, Umunyeshuli (No, student) - Oya , nta kazi (No, jobless) | |

**Part II: Sexual / Reproductive Health History**

| 1. Ujya ukora (Do you practice):  - Imibonano yo mu gitsina (Vaginal Sex) - Other(ibindi)_____________________   _____________________ | 1. Wakoze imibonano n’abantu bangahe, ikorewe  - Mu gitsina, muri iyi minsi 30 ishize? M_____F_____ - Mu kibuno, muri iyi minsi 30 ishize? M_____F_____ - Mu kanwa, muri iyi minsi 30 ishize? M_____F_____   (How many male/ female partners have you had Oral/  Vaginal/anal sex with in the last 30 days?) | | | |
| --- | --- | --- | --- | --- |
| 1. Ni kangahe wakoresheje agakingirizo mu mibonano mpuza bitsina mu mezi 3 ashize? (**Niba yarakoze imibonano ikorewe ku kibuno, andika mu bindi bisobanuro niba harakoreshejwe agakingirizo)**   (How often did you use condoms with vaginal sex in the last 3 months? If the client has had anal sex add in comments the use of condom)   - Buri gihe (Always) - Rimwe na rimwe (Sometimes) - Nta na rimwe (Never) - Sinigeze nyikora (I didn’t do sex in last 3 months) - Sinjya nyikora (I do not have vaginal sex) | | | | 1. Hashize iminsi ingahe kuva igihe utekereza ko wagize imibonano mpuzabitsina wanduriyemo izi ndwara? (How many days since you had sexual contact in which you suspect getting an STI from?) ___________ |
| **FOR FEMALE ONLY** | | | | |
| 1. Ufite abana bangahe bari munsi y’imyaka 18? (How many children under 18 years do you have) _________ **Niba igisubizo ari 0, singombwa kubaza Qn 21 (If answer is 0, no need to ask Qn21)** | | | 1. Urifuza kubyara abandi bana bangahe?   (How many more children would you like to have?) _________ | |
| 1. Uratwite? (Are you pregnant?)  - Yego (Yes) - Oya (No)   **Niba atwite jya ku kibazo cya 11(If yes skip to Qn 11)** | | 1. Urifuza gutwita? (Do you want to become pregnant?)  - Yego (Yes) - Oya (No) | | |
| 1. Niba udatwite ukaba utanifuza gutwita, hari uburyo bwo kuboneza urubyaro ukoresha? (If you are not currently pregnant and do not want to become pregnant, are you using any method to delay or avoid pregnancy?)  - Ntabwo (No method) - Ibinini (Pills) - Urushinge (Injectable) - Agapira ko mu kaboko (Hormonal Implant) - Agapira ko mu mura (IUD) - Kwifungisha burundu (Tubal ligation) - Agakingirizo (Condom) - Uburyo bwa kamere (natural method):   **⃝** Urunigi(collier)  **⃝** Kubara(calendar)  ⃝ Gusuzuma ururenda(cervical mucus)  **⃝** Kwiyakana(coit interrompu) | | 1. Haba hari uburyo yemeye gutangira gukoresha ? (Is there any method she accepts to use?)  - Oya (No) - Ibinini (Pills) - Urushinge (Injectable) - Agapira ko mu kaboko (Hormonal Implant) - Agapira ko mu mura (IUD) - Kwifungisha burundu (Tubal ligation) - Agakingirizo (Condom) - Uburyo bwa kamere (natural method):   **⃝** Urunigi(collier)  **⃝** Kubara(calendar)  ⃝ Gusuzuma ururenda(cervical mucus)  **⃝** Kwiyakana(coit interrompu) | | |

**Part III: Sexually Transmitted Infections**

| 1. Wigeze gusuzumwa ukanavurwa indwara zandurira mu mibonano mpuza bitsina (nka mburugu cyangwa imitezi mu mezi 12 ashize? (Have you ever been diagnosed and treated for an STI in the past 12 months?)  - Yego (Yes) - Oya (No) | 1. Uyu munsi hari ibimenyetso bikurikira waba ufite? (Do you currently have)  - Nta kimenyetso (No symptoms) - Kwishima ku gitsina (Genital itching) - Kwokerwa mu gihe wihagarika (Burning sensations when passing urine) - Ibintu by’uruzi bidasanzwe bica mu gitsina (Genital discharge) - Udusebe ku gitsina (Genital ulcer) - Ibintu by’uruzi cyangwa amashyira biva mu kibuno (Anal discharge) - Udusebe mu kibuno (Anal ulcer) - Udusununu mu kibuno (Anal warts) - Kumira ukababara (Sore Throat/Difficulty in swallowing) |
| --- | --- |
| 1. Hashize iminsi ingahe ufite ibyo bimenyetso (For how many days do you have those symptoms?) ___________ | |

**Part IV: HIV Testing History**

| 1. Wigeze wipimisha virusi itera SIDA? (Have you had an HIV test before?)  - Yego (Yes) - Oya (No)   **Niba ari oya jya ku kibazo cya 18(if No skip to Qn 19**) | 1. Ni ryari uheruka kwipimisha bwa nyuma?   Ukwezi n’umwaka  (When did you have your most recent HIV test?) (Month and Year)______/______ |
| --- | --- |
| 1. Igisubuzo giheruka wabonye ni ikihe? (What was   your most recent HIV test result?)   - Simbana na virusi itera SIDA (Negative) - Mbana na virusi itera SIDA (Positive) - Ntibyari bisobanutse (Doubtful/Undetermined) - Sinafashe igisubizo (Did not get result) - Sinshaka kukivuga (Do not want to disclose) | 1. Turagusaba ko washishikariza abo mukorana imibonano mpuza bitsina kuza kwipimisha. Urumva ubyifuza? (We recommend to bring your sex partners for testing as well. Would this be ok with you?)  - Yego (Yes) **(If yes give invitation)** - Oya (No) |

**Part V: PHYSICAL EXAM FINDINGS AND TEST REQUESTED**

| **VAGINAL DISCHARGE** | Yes | No | **SELF SWABS** |  | | Yes | No |  |  |  |
| --- | --- | --- | --- | --- | --- | --- | --- | --- | --- | --- |
| **ENDO DISCHARGE** | Yes | No | **VAGINAL SWABS** | |  | Yes | No | |  |  |
| **URETRAL DISCHARGE** | Yes | No | **ENDO SWABS** | |  | Yes | No | |  |  |
| **GENITAL ULCER** | Yes | No | **URETRAL SWABS** | |  | Yes | No | | |  |
|  |  |  | **URINE CT/NG**  **URINE MICROSCOPY**  **VL** | | Yes  Yes  Yes | | No  No  No | | | |
|  |  |  |  | |  | |  | | | |

**Part VI: LAB RESULTS/ DIAGNOSTIC**

| **TRICH** | Positive | Negative |  |  | |
| --- | --- | --- | --- | --- | --- |
| **CANDIDA** | Positive | Negative |  |  | |
| **BV** | Positive | Negative |  |  | |
| **GONORREA** | Positive | Negative |  |  | |
| **CHLAMIA** | Positive | Negative |  |  | |
| **HIV** | Positive | Negative | Doubtful | - Not Tested | |
| **RPR** | Positive | Negative | Titer | - Not Tested |  |
|  |  |  |  |  |  |

**Part VII: STI Treatment**

| No Treatment | Ciprofloxacin | Doxycyclin | Ceftriaxone | Erythromycin |
| --- | --- | --- | --- | --- |
| Penicillin | Acyclovir | Metronidazole | Tinidazole | Nystatine |
